# Improving Palliative Care and Quality of Life in Pancreatic Cancer Patients

**DOI:** 10.1089/jpm.2021.0187

**Published:** 2022-04-20

**Authors:** Vincent Chung, Virginia Sun, Nora Ruel, Thomas J. Smith, Betty R. Ferrell

**Affiliations:** ^1^ Department of Medical Oncology and Therapeutics Research, City of Hope, Duarte, California, USA.; ^2^ Division of Nursing Research and Education, Beckman Research Institute, City of Hope, Duarte, California, USA.; ^3^ Department of Computational and Quantitative Medicine, Beckman Research Institute, City of Hope, Duarte, California, USA.; ^4^ Department of Medicine, Division of Oncology, Johns Hopkins Sidney Kimmel Comprehensive Cancer Center, Baltimore, Maryland, USA.

**Keywords:** palliative care, pancreatic cancer, quality of life, symptoms

## Abstract

**Background:**

Pancreatic cancer patients often present with complications, which can impact treatment tolerance. Thus, symptom management is a vital component of treatment in addition to traditional chemotherapeutics. Concurrent palliative care with an emphasis on aggressive symptom management may sustain both clinical and patient-centered outcomes during treatment. The purpose of this article is to explore the impact of a concurrent palliative care intervention in patients with pancreatic cancer treated on phase I clinical trials.

**Materials and Methods:**

This is a secondary analysis of a National Cancer Institute (NCI)-funded randomized trial of an advanced practice nurse driven palliative care intervention for solid tumor patients treated on phase I clinical trials. Only pancreatic cancer patients were included in the analysis. Patients received two educational sessions around the quality of life (QOL) domains and completed the Functional Assessment of Cancer Therapy—General (FACT-G), patient-reported outcomes version of the common terminology criteria for adverse events (PRO-CTCAE), and the psychological distress thermometer at baseline, 4 and 12 weeks. Mixed model with repeated measures analysis was used to explore outcomes by study arm.

**Results:**

Of the 479 patients accrued to the study, 42 were diagnosed with pancreatic cancer (26 intervention, 16 usual care). A trend toward improvement in the physical, social, emotional, and functional FACT-G QOL subscales and psychological distress (baseline to 12 weeks) were observed for the intervention arm. Patients reported moderate severity in psychological and physical stress.

**Conclusions:**

In this secondary analysis, a nurse-led palliative care intervention may improve the QOL and psychological distress of pancreatic cancer patients. A phase III trial focused on patients with pancreatic cancer is needed to determine the effectiveness of the intervention.

## Introduction

Pancreatic adenocarcinoma is the fourth leading cause of cancer-related death in the United States. In 2021, it is estimated that 60,430 new cases were diagnosed with 48,220 estimated deaths.^1^ By 2030 pancreatic cancer will be the second leading cause of cancer-related death behind lung cancer. This is due to the limited effectiveness of available treatments. The two approved most commonly used regimens are FOLFIRINOX^2^ and gemcitabine plus nab-paclitaxel chemotherapy.^3^ Even with aggressive chemotherapy, median overall survival was less than one year. Therefore, palliative care and symptom management is an important modality of treatment for these patients.^4^

The complications of pancreatic cancer range from the physical through the psychological. Fatigue, abdominal pain, and weight loss are common symptoms of advanced disease leading to depression and social withdrawal. Obstructive jaundice leads to infections and hospitalizations delaying treatment and further worsening the patient's and family's anxiety. Given the complexity of the complications that occur with pancreatic cancer, multidisciplinary management with oncology, supportive medicine, psychology, nutrition, as well as other departments is essential to achieve the best outcomes.^5^

In early stages of cancer, surgery and radiation therapy can positively impact quality of life (QOL) by delaying progression. Most patients will have disease recurrence leaving chemotherapy or clinical trials as the only treatment options. Cytotoxic chemotherapy commonly causes nausea and vomiting, fatigue, loss of appetite, diarrhea, and neuropathy, which can complicate aggressive management to control the disease due to limitations in chemotherapy dose intensity. In the PRODIGE trial grade, 3/4 neutropenia occurred in 45.7% of patients. The FOLFIRINOX chemotherapy was administered every two weeks and required an infusion pump to administer the 5-fluorouracil over 46 hours. After 6 months of treatment, 31% of the patients had a definitive decrease in Global Health Status and QOL scale scores. Due to toxicities, the upper age limit of patients accrued to the trial was 76.^2^ With the median age of 69 in US patients, older nontrial patients might experience even more toxicity with aggressive chemotherapy.

The disease also causes physical and psychological complications. Jaundice due to biliary obstruction from the tumor can cause cholangitis leading to hospitalization. Pancreatic duct obstruction can lead to pancreatic insufficiency with chronic steatorrhea and protein-calorie malnutrition. The physical symptoms can exacerbate depression leading to the feeling of hopelessness. Without proper management of these complications, survival is compromised. Therefore, early identification of complications and referral to appropriate specialists is essential in the management of pancreatic cancer patients.

QOL is multidimensional incorporating one's perception of well-being based upon physical, psychological, social, and sexual domains. In pancreatic cancer patients, QOL is worse when compared to patients with other cancers. A review of 36 studies revealed that pancreatic cancer patients had decrements in QOL across all life domains with psychological distress being worse across all cancers. .^6,7^ With the limited number of therapies approved for the treatment of pancreatic cancer, patients frequently exhaust chemotherapy options and seek out early stage phase 1 clinical trials, which are complex studies evaluating the safety and tolerability of new drugs or combinations of drugs.^8^ Research tests evaluating the pharmacokinetic and pharmacodynamic actions of these drugs require multiple blood draws and potential biopsies with long hours spent in the hospital away from family. This adds additional stress on the patient with potential decrement of QOL.^9,10^

To examine the QOL-related outcomes for patients with pancreatic cancer enrolled on a phase I clinical trial, we conducted a subgroup analysis of 42 patients enrolled in a randomized trial testing the efficacy of a palliative care intervention. We previously reported that an interdisciplinary supportive care planning intervention was feasible in pancreatic cancer patients with over 80% of the patients reporting being highly satisfied with the intervention.^11^

## Materials and Methods

### Study design

The study evaluated the efficacy of a palliative care intervention in solid tumor patients on phase 1 clinical trials. Subjects were accrued at City of Hope and Sidney Kimmel Comprehensive Cancer Center at Johns Hopkins hospital. The purpose of the study was to evaluate the impact of a palliative care intervention on the patient's QOL, psychological distress, satisfaction with care, symptom management, and hospital resource utilization. The National Cancer Institute (NCI) funded study was unblinded and patients were randomized to either usual care or a palliative care intervention. The trial was approved by the Institutional Review Board at each site and registered with clinicaltrials.gov ID NCT01612598.^9^

### Eligibility criteria

Subjects were 21 years of age or older enrolling in a phase 1 solid tumor clinical trial. The type of trials enrolled included phase 1 first-time-in-human trials as well as phase 1 combination chemotherapy trials. Subjects were required to be fluent in English with no cognitive impairment. After consenting to the therapeutic trial, written informed consent for the palliative care intervention trial was obtained. Baseline data were collected on all subjects before the first dose of study treatment.

### Study procedures

**FIG. 1.**
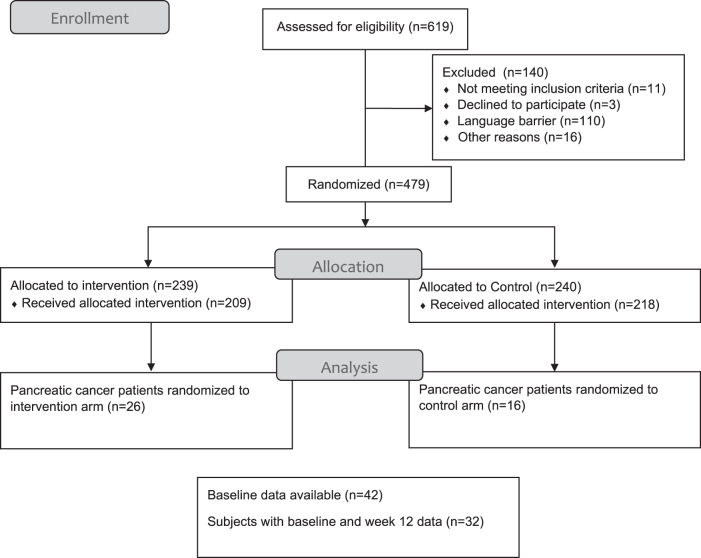
CONSORT diagram—NCT01612598: palliative care intervention in patients with solid tumors participating in phase I clinical trials. Four hundred seventy-nine subjects with advanced solid tumors were randomized to either intervention or control. We analyzed the subset of 42 subjects with pancreatic cancer.

Subjects randomized to usual care received standard treatment for patients enrolled in the phase 1 clinical trial. For subjects in the palliative care intervention group, a care plan was created by the advanced practice nurse based upon data from the baseline evaluation. An interdisciplinary meeting of the study investigators, including a physician, nurse, chaplain, and social worker, discussed management of symptoms that were revealed in the baseline survey. The treating physician was invited to participate in the discussion, however, if unavailable, written recommendations were sent to the patient's physician. These reports included recommendations to refer to appropriate specialists and the treating physician would either submit the consult or manage directly. For example, if a patient is complaining of severe pain from metastases, radiation oncology may be appropriate for palliation, but not yet explored by the treating physician yet. This intervention may help to facilitate care for a busy practitioner by providing two teaching sessions with the advanced practice nurse utilizing standardized teaching materials addressing symptomatic QOL concerns. Follow-up evaluations occurred at 4 and 12 weeks. This was a prospective, randomized clinical trial powered to detect differences in psychological distress, symptom intensity, and symptom severity in patients receiving usual care or the intervention with the advanced practice nurse. QOL and related metrics were included as secondary endpoints. The study accrued 479 patients with 42 having pancreatic cancer (Fig. 1). Three time points were used for prediction of outcome measures. The control group was offered participation in the Palliative Care Intervention (PCI) program after week 12. The main results were reported and showed the nurse-delivered intervention improved some QOL outcomes and distress.^9^ Here, we report on the pancreatic cancer subset.

### Psychological distress scale

The psychological distress scale is a single item asking patients to rate their distress on a scale of 0 (none) to 10 (extreme distress).^12^

### Functional Assessment of Cancer Therapy—General

The Functional Assessment of Cancer Therapy—General (FACT-G) is a well-established validated QOL scale consisting of 27 items rated on a scale of 0–4. Subscales include physical well-being, social/family well-being, emotional well-being, functional well-being, and overall QOL. This is scored on a five-point scale ranging from 0 to 4 with 0 being “not at all” and 4 being “very much.” The highest possible score for the emotional well-being subscale is 24. The other three subscales score up to 28. The total FACT-G score can range from 0 to 108 with higher scores indicating better QOL.^13^

### PRO-questionnaire

The patient-reported outcomes version of the common terminology criteria for adverse events (PRO-CTCAE) library consists of 78 adverse events representing 124 distinct items. Forty-five items that deemed relevant to the pancreatic study population based upon the study team's opinion were selected for descriptive analysis. The trial participants completed the questionnaires before clinic appointments.^14^

### Statistical methods

This study was designed as a randomized, prospective, longitudinal two-group experiment, powered to detect significant group differences in coprimary endpoints psychological distress, symptom intensity, and symptom severity, while QOL and other related metrics were included as secondary endpoints.

Data were analyzed according to intent to treat. Descriptive statistics were generated for all data collected, instrument scores were calculated according to instructions, and distributions were examined. Baseline data for the FACT-G and Psychological Distress tools were used to estimate reliability for this study's subjects for this instrument. Mixed model with repeated measures (MMRM) analysis—with three timepoints: baseline, 4 weeks, 12 weeks—was used to explore outcome measures, and to determine significance of effects from treatment arm as well as interaction effects between treatment arm and evaluation time point.

Pancreatic cancer patients who received at least part of the intervention and had at baseline and either week 4 or 12 assessments were included in the MMRM analysis (*n* = 32). Ten patients who only completed baseline assessments were therefore excluded from this analysis. Differences in least squares means estimates between baseline and 12 weeks were used to calculate change in the overall FACT-G Index and subscale scores, and Psychological Distress. Statistical significance was determined with *t*-tests, using Tukey–Kramer adjustments in pairwise comparisons of least square means across subgroups in MMRM. A type I error of 0.05 was used as a threshold for statistical significance. SAS 9.4^®^ was used to conduct analyses.

## Results

### Demographic data

Baseline patient characteristics for the 42 pancreatic cancer patients are presented in Table 1.

**Table 1.** Patient Demographics

Baseline demographic data	All pancreatic patients (*n* = 42)	Experimental arm (*n* = 26)	Control arm (*n* = 16)
Treatment arm, *n* (%)			
Experimental	26 (61.9)	26 (100.0)	0 (0.0)
Control	16 (38.1)	0 (0.0)	16 (100.0)
Age, mean (range)	61.8 (21.0–83.0)	60.0 (21.0–83.0)	64.7 (44.0–78.0)
Gender, *n* (%)			
Female	18 (42.9)	11 (42.3)	7 (43.8)
Male	24 (57.1)	15 (57.7)	9 (56.3)
Race/Ethnicity, *n* (%)			
African American	5 (11.9)	3 (11.5)	2 (12.5)
Asian	5 (11.9)	4 (15.4)	1 (6.3)
Caucasian	28 (66.7)	16 (61.5)	12 (75.0)
Hispanic Latino	1 (2.4)	1 (3.8)	0 (0.0)
Native Hawaiian	1 (2.4)	1 (3.8)	0 (0.0)
Mixed race	2 (4.8)	1 (3.8)	1 (6.3)
Education, *n* (%)			
Did not complete high school	1 (2.4)	1 (3.8)	0 (0.0)
High school	9 (21.4)	4 (15.4)	5 (31.3)
College	20 (47.6)	12 (46.2)	8 (50.0)
Graduate/professional school	11 (26.2)	8 (30.8)	3 (18.8)
Unknown	1 (2.4)	1 (3.8)	0 (0.0)
Religion, *n* (%)			
None	9 (21.4)	5 (19.2)	4 (25.0)
Catholic	8 (19.0)	6 (23.1)	2 (12.5)
Jewish	1 (2.4)	0 (0.0)	1 (6.3)
Protestant	20 (47.6)	12 (46.2)	8 (50.0)
Other	4 (9.5)	3 (11.5)	1 (6.3)
Marital status, *n* (%)			
Never married	3 (7.1)	3 (11.5)	0 (0.0)
Widowed	4 (9.5)	2 (7.7)	2 (12.5)
Divorced/separated	1 (2.4)	1 (3.8)	0 (0.0)
Married/living with partner	34 (81.0)	20 (76.9)	14 (87.5)
Employment status, *n* (%)			
Employed full time	7 (16.7)	7 (26.9)	0 (0.0)
Employed part time	4 (9.5)	1 (3.8)	3 (18.8)
Homemaker	3 (7.1)	2 (7.7)	1 (6.3)
Retired	23 (54.8)	12 (46.2)	11 (68.8)
Unemployed	5 (11.9)	4 (15.4)	1 (6.3)
Family income, *n* (%)			
$10,001–$20,000	1 (2.4)	1 (3.8)	0 (0.0)
$20,001–$30,000	2 (4.8)	2 (7.7)	0 (0.0)
$30,001–$40,000	5 (11.9)	3 (11.5)	2 (12.5)
$40,001–$50,000	4 (9.5)	3 (11.5)	1 (6.2)
Greater than $50,000	29 (69.0)	16 (61.5)	13 (81.2)
Unknown	1 (2.4)	1 (3.8)	0 (0.0)
Current or previous surgical procedure, *n* (%)	18 (42.9)	11 (42.3)	7 (43.8)
Current or previous chemotherapy, *n* (%)	32 (76.2)	18 (69.2)	14 (87.5)
Current or previous radiation therapy, *n* (%)	12 (28.6)	8 (30.8)	4 (25.0)
Tried alternative therapies, *n* (%)	9 (21.4)	5 (19.2)	4 (25.0)
Number of comorbidities, median (range)	1.5 (0–6)	1 (0–6)	2 (2–5)

A majority of patients enrolled were male (57.1%) with a median age of 62. One-third (33%) of the patients enrolled were from minority groups. Over 73% of the patients participating were highly educated with at least a college degree. 76.2% of patients received prior chemotherapy, while 21.4% of patients tried alternative treatments.

### Patient-reported outcomes

Patient reported symptom frequency and severity were scored from 0 (none) to 4 (very severe). Using the validated FACT-G questionnaire, we evaluated 27 questions determined to be important for pancreatic cancer patients (Table 2). This measures the four domains of health-related QOL in cancer patients (physical, social, emotional, and functional well-being).

**Table 2.** Summary of Functional Assessment of Cancer Therapy—General Questions at Baseline

FACT-G and psychological distress tools	All pancreatic patients (*n* = 42)	Experimental (*n* = 26)	Control (*n* = 16)
Lack energy	2.2 (1.0)	2.2 (1.0)	2.2 (0.8)
Have nausea	3.4 (0.9)	3.2 (0.9)	3.6 (0.7)
Trouble meeting family needs	3.1 (1.1)	3.0 (1.2)	3.3 (0.9)
Have pain	2.3 (1.1)	2.1 (1.1)	2.6 (1.2)
Bothered by side effects	3.3 (0.8)	3.4 (0.9)	3.3 (0.9)
Feel ill physically	3.0 (1.0)	2.9 (1.1)	3.2 (0.8)
Forced in bed	3.2 (1.0)	3.1 (1.1)	3.4 (0.8)
Close to friends	3.6 (0.7)	3.5 (0.8)	3.7 (0.7)
Emotional support from family	3.9 (0.4)	3.9 (0.3)	3.8 (0.5)
Support from friends	3.6 (0.7)	3.6 (0.6)	3.6 (0.7)
Family accepted illness	3.6 (0.9)	3.5 (1.0)	3.7 (0.7)
Satisfied with communication about illness	3.7 (0.7)	3.6 (0.8)	3.8 (0.5)
Feel close to partner	3.9 (0.5)	3.9 (0.3)	3.8 (0.7)
Satisfied with sex life	2.1 (1.7)	2.0 (1.7)	2.1 (1.6)
Feel sad	2.5 (1.1)	2.3 (1.2)	2.7 (0.8)
Coping with illness	3.0 (1.1)	2.8 (1.2)	3.3 (0.8)
Losing hope with fighting illness	2.8 (1.2)	2.8 (1.2)	2.8 (1.1)
Feel nervous	2.5 (1.1)	2.4 (1.1)	2.8 (0.9)
Worry about dying	2.4 (1.4)	2.3 (1.6)	2.6 (1.2)
Worry condition will get worse	1.5 (1.3)	1.5 (1.4)	1.6 (1.1)
Able to work	2.5 (1.2)	2.4 (1.3)	2.6 (1.0)
Work is fulfilling	2.6 (1.3)	2.5 (1.3)	2.7 (1.3)
Able to enjoy life	2.6 (1.1)	2.4 (1.1)	2.9 (0.9)
Accepted illness	2.9 (1.2)	2.7 (1.1)	3.1 (1.3)
Sleeping well	2.8 (1.3)	2.5 (1.3)	3.3 (1.1)
Enjoying things for fun	2.5 (1.3)	2.2 (1.5)	2.9 (1.0)
Content with quality of life	2.2 (1.4)	2.0 (1.5)	2.4 (1.2)

Mean (SD) of 27 FACT-G questions scored 0–4.

FACT-G, Functional Assessment of Cancer Therapy—General; SD, standard deviation.

Many of the concerns observed related to the side effects from prior treatment. Patients typically had residual side effects from therapy, which impacted their QOL. With advanced disease, physically feeling ill resulted in more time in bed. Nausea was also a problem with many of the patients either due to the chemotherapy treatments or obstructive symptoms from the tumor. Worrying about the illness was significant leading to losing hope and trouble coping with illness, but there was strong emotional support from family as well as friends. The overall FACT-G score at baseline show low emotional and functional well-being scores across the groups (Table 3). This resulted in a low overall FACT-G score of 77.7 for all pancreatic cancer patients. The Psychological Distress Scale rates distress from 0 to 10 with a score of 5 or higher indicating a need for intervention. Overall, in our study, the psychological distress score was 3.9 for all pancreatic cancer patients.

**Table 3.** Overall FACT-G Score and Psychological Distress, and FACIT-SP12 at Baseline

FACT-G and psychological distress tools	All pancreatic patients (*n* = 42)	Experimental (*n* = 26)	Control (*n* = 16)
Overall FACT-G score (0–108, higher scores better)	77.7 (16.4)	75.2 (18.0)	81.7 (12.8)
Physical well-being subscale (0–28)	20.6 (4.9)	20.0 (5.6)	21.5 (3.6)
Social well-being subscale (0–24)	24.4 (3.2)	24.3 (3.4)	24.7 (2.9)
Emotional well-being subscale (0–28)	14.7 (5.1)	14.2 (5.7)	15.6 (3.9)
Functional well-being subscale (0–28)	18.0 (7.1)	16.8 (7.4)	19.9 (6.1)
Psychological Distress (0–10, lower scores better)	3.9 (2.7)	4.1 (3.1)	3.5 (2.0)
FACIT-SP12 score (0–48, higher scores better)	37.0 (9.8)	37.2 (10.5)	36.8 (8.8)

Overall FACT-G score 0–108 possible points with higher scores being better.

Psychological distress is scored 0–10 with lower scores being better.

FACIT-SP12, Functional Assessment of Chronic Illness Therapy-Spiritual Well-Being 12.

Table 4 summarizes the differences of least squares means (and standard errors) from baseline to week 12 for (1) all pancreatic patients, (2) experimental only, (3) control only, and (4) experimental versus control, to demonstrate the effectiveness of the intervention with respect to the control group. The MMRM analysis takes into account the week 4 data and covers all timepoints from baseline to week 12.

**Table 4.** Mixed Model with Repeated Measures Differences of Lease Squares Means (Standard Error) in Functional Assessment of Cancer Therapy—General (with Subscales) and Psychological Distress, from Baseline to Week 12

Instrument	All pancreatic patients (*n* = 32)	Experimental (*n* = 18)	Control (*n* = 14)	Experimental vs. control^a^
	BL to W12	BL to W12	BL to W12	
FACT-G Index (QOL)	0.10 (3.44)	7.46 (4.66)	−7.25 (5.07)	4.38 (4.33)
Physical well-being	−0.46 (1.18)	−0.33 (1.59)	−0.60 (1.73)	0.26 (1.47)
Social well-being	−1.67 (0.61)^*^	0.15 (0.83)	−3.49 (0.91)^**^	0.91 (0.94)
Emotional well-being	1.44 (1.08)	4.07 (1.46)	−1.19 (1.59)	1.68 (1.56)
Functional well-being	0.62 (1.39)	3.30 (1.88)	−2.07 (2.04)	1.52 (1.86)
Psychological Distress	−0.76 (0.65)	−1.80 (0.87)	0.29 (0.95)	−1.26 (0.72)
FACIT-SP12 Index	−1.0 (1.31)	0.68 (1.70)	−2.67 (0.76)	2.11 (3.34)

^a^ Experimental versus control column: demonstrates effect of PCI versus control group over time.

*p*-Value result is from *t*-test with Tukey–Kramer adjustment are denoted with ^*^(*p* < 0.05), or ^**^(*p* < 0.01).

BL, baseline; PCI, Palliative Care Intervention; QOL, quality of life; W12, week 12.

This showed in the experimental arm versus control an improvement in the FACT-G index for physical well-being, social well-being, emotional well-being, and functional well-being. Psychological distress also improved in the experimental arm. In the Functional Assessment of Chronic Illness Therapy Spiritual Well-being scale, the experimental group showed a slight improvement over the course of 12 weeks with intervention while the control group decreased over the 12 weeks. Statistical significance is only noted in the social well-being subscales. However, since the direction in the results consistently favors the experimental arm, one explanation could be that the lack of statistical significance may be the result of the small sample size in our subset analysis.

## Discussion

The diagnosis of pancreatic cancer is associated with stressors affecting QOL and the complexity of an experimental clinical trial adds to that stress. This secondary analysis from an NCI-funded randomized clinical trial provides additional QOL data on pancreatic cancer patients enrolling in phase 1 clinical trials. The concerns of patients shifted as they enrolled in phase 1 clinical trials. Initially, when patients are diagnosed, physical symptoms such as pain and lack of energy are the major concerns. Uncertainty about the burden on family and friends adds to the stress, which can lead to depression. Early intervention with palliative care can have a positive impact on treatment potentially leading to longer survival by allowing patients to stay on treatment.^15,16^ The physical and psychological symptoms improve with control of disease. However, when the cancer becomes refractory, we observed a shift in the distress of our patients enrolled in the trial. At this point, patients have been heavily pretreated with multiple chemotherapy regimens. Even though they are well versed in managing the toxicities of treatment, they are now faced with the cumulative toxicities of the chemotherapy. Neuropathy can impair routine daily activities making them more dependent on family and friends. Refractory cancer increases sadness and anxiety about dying.^17^ Patients are physically ill spending more time in bed. Interestingly, we observed bonds with family and friends were stronger at this point potentially due to more resources being available for patients enrolling in phase 1 trials. Also, there appears to be a dose-response aspect to PC for pancreas cancer patients; the earlier and more frequent the PC visits, the less hospitalization and aggressive care at the end of life.^18,19^

There are limitations to this small subset analysis. With only 26 patients in the experimental arm and 16 in the control arm, definitive conclusions cannot be made. Also, there is a selection bias for patients seeking phase 1 clinical trials at comprehensive cancer centers. Many of the patients enrolled were of higher socioeconomic status as well as education level. Phase 1 clinical trial eligibility criteria commonly limit accrual to good performance status patients Eastern Cooperative Oncology Group (ECOG) 0–1. clinicaltrials.gov lists 4052 early-phase interventional studies being conducted in the United States. Being able to research these studies to find an appropriate clinical trial requires education and resources. This is reflected in the demographic data and highlights the importance of outreach to communities with limited resources. Second, although outcome measures queried patients on their perception of social support, the randomized trial did not include family caregivers. We have previously shown that a nurse-led palliative care intervention improved social well-being, distress, and reduced burden for lung cancer family caregivers.^20^ Given the high initial symptom burden in patients with pancreatic cancer, early support for family caregivers may be essential to sustaining QOL.^6^

## Conclusions

Palliative care is an integral part in the management of pancreatic cancer patients. We have shown a positive impact of a palliative care intervention improving QOL measures across pancreatic cancer patients enrolled in phase 1 trials. Temel et al. previously demonstrated an improvement in survival for lung cancer patients receiving early palliative care.^21^ FACT-L scores in the Temel study improved in the intervention arm indicating improved QOL, which correlated with improved survival. Two other recent studies showed that monitoring and fixing symptoms in advanced cancer patients leads to substantially improved survival. In pancreatic cancer patients, we hypothesize that early palliative care will impact survival.^22,23^ To answer that question, we have designed a randomized phase 3 clinical trial of a primary palliative care intervention to improve QOL in metastatic pancreatic cancer patients. S2016 will be conducted at SWOG and NCORP sites across the country to provide access to rural communities. With the shortage of palliative care physicians, we designed a scalable approach to provide palliative care to a population in need. With centrally located advanced practice nurses, needs can potentially be identified early to guide interventions by the local teams to positively impact our patients and family caregivers QOL.
